# Diverse Roles of Cellular Senescence in Skeletal Muscle Inflammation, Regeneration, and Therapeutics

**DOI:** 10.3389/fphar.2021.739510

**Published:** 2021-09-06

**Authors:** Yuki Saito, Takako S. Chikenji

**Affiliations:** ^1^ Department of Anatomy, Sapporo Medical University School of Medicine, Sapporo, Japan; ^2^ Department of Health Sciences, School of Medicine, Hokkaido University, Sapporo, Japan

**Keywords:** senescence, skeletal muscle, chronic inflammation, aging, muscle regeneration, muscle stem cells, FAPs, fibrosis

## Abstract

Skeletal muscle undergoes vigorous tissue remodeling after injury. However, aging, chronic inflammatory diseases, sarcopenia, and neuromuscular disorders cause muscle loss and degeneration, resulting in muscular dysfunction. Cellular senescence, a state of irreversible cell cycle arrest, acts during normal embryonic development and remodeling after tissue damage; when these processes are complete, the senescent cells are eliminated. However, the accumulation of senescent cells is a hallmark of aging tissues or pathological contexts and may lead to progressive tissue degeneration. The mechanisms responsible for the effects of senescent cells have not been fully elucidated. Here, we review current knowledge about the beneficial and detrimental effects of senescent cells in tissue repair, regeneration, aging, and age-related disease, especially in skeletal muscle. We also discuss how senescence of muscle stem cells and muscle-resident fibro-adipogenic progenitors affects muscle pathologies or regeneration, and consider the possibility that immunosenescence leads to muscle pathogenesis. Finally, we explore senotherapy, the therapeutic targeting of senescence to treat age-related disease, from the standpoint of improving muscle regeneration.

## Introduction

Cell senescence was first described more than 50 years ago (Hayflick and Moorhead, 1961). Hayflick and Moorhead observed that normal human fibroblasts have a finite proliferative capacity in culture. They termed the cell cycle arrest at the exhaustion of this capacity “replicative senescence”; the word “senescence” is derived from the Latin word *senex,* meaning “old.” Subsequently, it was recognized that cellular senescence arises from telomere shortening, which is associated with chromosomal instability; accordingly, senescence was viewed as a tumor suppressor mechanism (Serrano et al., 1997; Lowe et al., 2004). Later studies revealed the physiological importance of cellular senescence beyond its tumor suppressor functions in processes such as wound healing (Jun and Lau, 2010; Demaria et al., 2014), embryonic development (Storer et al., 2013), and tissue repair and regeneration (Krizhanovsky et al., 2008; Muñoz-Espín and Serrano, 2014). On the other hand, cellular senescence also contributes to organismal aging and related diseases (Song et al., 2020). Senescent cells are metabolically active and secrete a variety of factors: inflammatory cytokines and chemokines collectively termed the senescence-associated secretory phenotype (SASP), which can induce chronic inflammation (Coppé et al., 2008; Gorgoulis et al., 2019, Birch 2020). A recent study showed that senescent cells can induce other non-senescent cells to undergo senescence by juxtacrine or paracrine effects, including the SASP, a phenomenon known as secondary senescence (Admasu 2021). In addition, cellular senescence can occur in post-mitotic cells, such as neurons and muscle cells (Zglinicki 2021). These discoveries suggested that senescent cells can have a widespread impact on various tissue and pathologies. Therefore, senolytic agents, which eliminate accumulated senescent cells, have attracted a great deal of attention as potential treatments for age-related diseases, which are frequently associated with chronic inflammation (Bussian et al., 2018; Xu et al., 2018; Hickson et al., 2019; Justice et al., 2019). Multiple aspects of cellular senescence are involved in skeletal muscle physiology and disease (Kudryashova et al., 2012; Saito et al., 2020; Sugihara et al., 2020), raising the question of whether fundamentally different mechanisms underlie the beneficial and detrimental effects of senescence in skeletal muscle. This review focuses on how senescent cells are involved in skeletal muscle physiology and pathology and how senolytics or pro-senescent therapies (including exercise) could be used to treat diseases of skeletal muscle.

## Mechanisms of Senescence

Cellular senescence is an adaptive response induced by multiple physiological and pathological stresses that results in irreversible cell cycle arrest (Muñoz-Espín and Serrano, 2014; van Deursen, 2014; Gorgoulis et al., 2019; Borghesan et al., 2020). Senescence provides a defense mechanism that limits tumorigenesis to maintain tissue homeostasis and allow tissue remodeling via removal of damaged senescent cells (Muñoz-Espín and Serrano, 2014). However, permanent accumulation of senescent cells is a major cause of age-related disease and chronic inflammation. Leonard Hayflick and Paul Moorhead found that human fibroblasts have a finite *in vitro* proliferative capacity (Hayflick and Moorhead, 1961), and subsequent work showed that replicative senescence is caused by the shortening of the telomeres at the ends of chromosomes, which triggers the DNA damage response (DDR) and causes cell cycle arrest (Sedelnikova et al., 2004; Campisi and d'Adda di Fagagna, 2007). Similarly, cellular senescence can arise due to DNA damage from various stresses, including radiation (Le et al., 2010), oncogene activation (Di Micco et al., 2006), high levels of reactive oxygen species (ROS) (von Zglinicki, 2002), mitochondrial dysfunction (Chapman et al., 2019), mechanical stress (Xing et al., 2010), protein aggregation (Johmura et al., 2021), failure of protein removal due to diminished autophagy (García-Prat et al., 2016), and inflammatory cytokines and growth factors (Beyne-Rauzy et al., 2004; Hubackova et al., 2012). These stresses activate DDR components including ATR, ATM, and p53, which promote activation of cyclin-dependent kinase (CDK) inhibitors such as p16^INK4a^ (*CDKN2A*) and p21^WAF1/Cip1^(*CDKN1A*) (Gorgoulis et al., 2019). Defects in ribosome biogenesis and derepression of retrotransposons also contribute to cell cycle arrest in senescent cells (Lessard et al., 2018; De Cecco et al., 2019). To date, however, no specific markers of the cell cycle have been identified in senescent cells. For example, p16^INK4a^ is also expressed in non-senescent cells (Sharpless and Sherr, 2015) and is not expressed in all senescent cells (Hernandez-Segura et al., 2017). Furthermore, senescence induced by E2F3 activation or c-Myc inhibition is DDR-independent and involves p16^INK4a^ and p19ARF (Lazzerini Denchi et al., 2005). Another DDR-independent inducer of cellular senescence is BRAF (V600E), which activates senescence through a metabolic mechanism involving upregulation of mitochondrial pyruvate dehydrogenase (Kaplon et al., 2013; van Deursen, 2014). Senescent cells exhibit characteristic morphological and physiological changes associated with this condition. In other words, senescent cells become hypertrophied and flattened *in vitro*, and the nuclear envelope is incomplete due to reduced expression of lamin B1 (González-Gualda et al., 2021). Accumulation of senescence-associated β-galactosidase (SA-β-gal) due to changes in lysosomal activity is another characteristic of cellular senescence (Gorgoulis et al., 2019). Chromatin rearrangement, especially the formation of senescence-related heterochromatin foci (SAHFs), is a frequently observed biomarker in oncogene-induced senescent (OIS) cells. SAHFs contain histone H3 methylated on lysine 9 (H3K9Me) (Braig et al., 2005), heterochromatin protein 1 (HP1), and histone H2A variant H2AX phosphorylated on Ser139 (γH2AX), and thus can be used as indicators of DNA damage to assess senescence (Dungan et al., 2020). To date, however, specific and sensitive markers of senescent cells have not been identified. Consequently, combinations of biomarkers, such as nuclear (p16^INK4a^, p21^WAF1/Cip1^, Ki67, γH2AX), cytoplasmic (SA-β-gal), SASP, context, and cell-type-specific markers, are generally used to define the presence of senescence (Coppé et al., 2008; Childs et al., 2015; Gorgoulis et al., 2019).

Senescent cells limit their own proliferation but remain metabolically active, secreting a variety of factors: inflammatory cytokines such as IL-6, IL-8, and TNF-⍺; chemokines; growth factors such as TGFβ; matrix metalloproteinases (MMPs); and micro-RNAs. Collectively, these secreted factors are referred to as the SASP (Coppé et al., 2008). The SASP is considered a hallmark of cellular senescence, and some of the secreted factors exert various autocrine/paracrine effects on the microenvironment of surrounding tissues. The SASP has both beneficial and detrimental consequences, depending on the context. For example, the SASP recruits immune cells to initiate tissue repair through removal of damaged cells (Krizhanovsky et al., 2008; Chikenji et al., 2019) but is also associated with angiogenesis and ECM remodeling, which may promote tumor cell progression (Gonzalez-Meljem et al., 2018; Levi et al., 2020). Although the SASP regulates beneficial effects such as developmental senescence (Muñoz-Espín et al., 2013; Storer et al., 2013) and wound healing (Demaria et al., 2014), it also contributes to the pathology of chronic inflammation (Franceschi and Campisi, 2014). The composition of the SASP varies depending on senescence trigger and cell type. For example, mitochondria dysfunction causes a distinct senescence response, termed mitochondrial dysfunction–associated senescence (MiDAS). MiDAS is associated with lower NAD+/NADH ratios, which both cause growth arrest and prevent the IL-1–associated SASP through AMPK-mediated p53 activation (Wiley et al., 2016). A recent analysis demonstrated that soluble SASP factors, including exosomes, differ markedly among different senescence triggers and distinct cell types, and also identified common SASP factors representing the “core SASP” (Basisty et al., 2020). The authors also found that several SASP factors, including growth/differentiation factor 15 (GDF15), stanniocalcin 1 (STC1), and serine protease inhibitors (SERPINs), correlated with age in plasma from a human cohort (Basisty et al., 2020). The composition and strength of the SASP are dynamic, changing at intervals after senescence induction (Hernandez-Segura et al., 2017). The dynamic and complex nature of the SASP is likely critical to the diverse biological functions associated with senescence.

### Beneficial vs. Detrimental Senescence and Therapeutic Targeting of Senescence (Senotherapy)

Cellular senescence plays roles in diverse processes ranging from embryonic development to wound healing, tissue repair, regeneration, cancer, aging, and age-related disease. During normal development, senescent cells are regulated by TGFβ/SMAD and PI3K/FOXO pathways and express the SASP to recruit immune cells, which can remodel tissue through cell clearance (Muñoz-Espín et al., 2013; Storer et al., 2013). Senescence also prevents tumorigenesis. Specifically, oncogene activation induces cell cycle inhibitors such as p16^INK4a^ and p53, which offset oncogenic signaling and cause cells to enter senescence, thereby preventing tumorigenesis (Muñoz-Espín and Serrano, 2014). Furthermore, SASP-mediated inflammation can help recruit tumor-targeting immune cells, thus providing a barrier against tumor formation (Rao and Jackson, 2016). Therefore, the pro-senescence approach has been proposed as a cancer treatment protocol (Nardella et al., 2011). Cellular senescence has also been proposed to ameliorate the effect of skin scarring (Jun and Lau, 2010), oral submucous fibrosis (Pitiyage et al., 2011), liver fibrosis (Krizhanovsky et al., 2008), and renal fibrosis (Wolstein et al., 2010) as well as promote cardiac regeneration (Feng et al., 2019). Most types of senescent cells are activated fibroblasts/mesenchymal cells, and deletion of the senescent cells promotes fibrosis. For example, skin fibroblast senescence is induced by the extracellular matrix protein CCN1 (also known as CYR61), which is associated with the expression of pro-inflammatory cytokines and antifibrotic MMPs (Jun and Lau, 2010). Cyr61-deficient mice do not activate senescence in skin fibroblasts that promote cutaneous healing, leading to exacerbated fibrosis (Jun and Lau, 2010). As in skin fibrosis and recovery, liver fibrosis is also limited by senescence of hepatic stellate cells (HSC). Activated HSCs upregulate p53, p21^WAF1/Cip1^, and p16^INK4a^, which are associated with the SASP. The SASP attracts immune cells, promotes clearance of senescent HSCs by NK cells, and eliminates fibrotic scars (Krizhanovsky et al., 2008). Furthermore, senescence of fibroadipogenic progenitors (FAP) limits skeletal muscle fibrosis and regulates tissue repair after injury (Saito et al., 2020).

Although cellular senescence has beneficial effects by promoting the clearance of senescent cells, chronic accumulation of senescent cells in aged individuals promotes age-related disease and tissue dysfunction. Accumulation of senescent cells is observed particularly in age-associated chronic inflammatory diseases, such as chronic kidney disease (Docherty et al., 2019), idiopathic pulmonary fibrosis (Schafer et al., 2017), diabetes (Palmer et al., 2019), atherosclerosis (Wang and Bennett, 2012), sarcopenia (Sousa-Victor et al., 2014), osteoarthritis (Coryell et al., 2021), osteoporosis (Farr and Khosla, 2019), and obesity (Ogrodnik et al., 2019). Furthermore, higher levels of senescent cells are observed in neurodegenerative disorders such as Alzheimer’s disease and Parkinson’s disease (Riessland et al., 2019; Zhang et al., 2019).

It remains unclear why senescent cells accumulate. Apoptotic resistance of senescent cells appears to contribute to tissue dysfunctions (Wang, 1995). Senescent cells may prevent their own clearance through protective anti-apoptotic pathways by upregulating BCL-2 family members (Zhu et al., 2015). Impaired elimination of senescent cells by the immune system also results in the accumulation of senescent cells. Surveillance of senescent cells is performed by various immune cell types, including macrophages, neutrophils, natural killer (NK) cells, and CD8^+^ T cells, depending on the pathophysiological situation (Ovadya et al., 2018; Chikenji et al., 2019). HLA-E, a non-classical MHC-class Ib molecule, inhibits immune responses against senescent dermal fibroblasts by interacting with the inhibitory receptor NKG2A expressed on NK cells and highly differentiated CD8^+^ T cells (Pereira et al., 2019).

Approaches for therapeutically targeting senescence to improve age-related disease, known as senotherapy, have developed rapidly. Senolytics are senotherapies aimed at selective elimination of senescent cells through programmed cell death, including apoptosis. The molecular targets of senolytics include PI3K/AKT and p53/p21 (Kim et al., 2019), Bcl-2/Bcl-x family (Zhu et al., 2015, 2016; Chang et al., 2016; Yosef et al., 2016), p53/Mdm2 (He et al., 2020), and p53/FOXO4 (Zhang et al., 2020). Among senolytics, dasatinib (D)/quercetin (Q) combination therapy has been extensively clinically researched; its potential molecular targets are PI3k/AKT/mTOR, BCL-xL, ephrins, p21, and PAI-2 (Zhu et al., 2015; Cavalcante et al., 2020). Clinical trials are planned, ongoing, or completed for idiopathic pulmonary fibrosis, Alzheimer’s disease, chronic kidney disease, frailty, and skeletal aging (ClinicaTtrials.gov identifiers: NCT02874989, NCT04785300, NCT04685590, NCT02848131, NCT03675724, NCT03430037, and NCT04313634).

While senolytics aim at selective elimination of senescent cells, another approach is to target the SASP (so-called “senomorphic” therapy) (Gorgoulis et al., 2019). Rapamycin is considered a senomorphic drug that inhibits mTOR and promotes autophagy, which reverses senescence (García-Prat et al., 2016). Metformin is another senomorphic drug that activates AMPK and regulates mTOR (Li et al., 2020); however, metformin also promotes beneficial senescence, which enhances the anticancer effect (Hu et al., 2020).

## Senescence in Skeletal Muscle

Several studies have reported a relationship between senescence and muscle diseases, including aging. In skeletal muscle tissue of aged mice, the mRNA expression of *Cdkn2a*, *Cdkn1a*, *Trp53*, *Gadd45a*, *Il6*, *Serpine1*, *Mmp1*, and *Mmp3* is elevated, and muscle mass and muscle functions are reduced (Edwards et al., 2007; Baker et al., 2016; Solovyeva et al., 2021). In humans, skeletal muscle tissue from older women (65–71 years old) expresses higher levels of *CDKN1A* than skeletal tissue from young women (20–29 years old) (Welle et al., 2004). Several potential mechanisms of muscle cell senescence–mediated, aging-associated muscle loss (sarcopenia) were proposed based on an *in vitro* study using C2C12 cells, a murine myoblast cell line (Alcalde-Estévez et al., 2020; Mijares et al., 2021; Moustogiannis et al., 2021). These studies revealed that elevated intracellular Ca^2+^ concentration or endothelin-1 receptor in C2C12 cells induces cellular senescence, and senescent C2C12 cells increase the expression of apoptotic, atrophic, and inflammatory factors, which might in turn induce sarcopenia mediated by muscle cell senescence (Alcalde-Estévez et al., 2020; Mijares et al., 2021; Moustogiannis et al., 2021). On the other hand, recent *in vivo* analysis of human skeletal muscle revealed that there is no difference in the abundance of γH2AX-positive myonuclei between young individuals (21–30 years old) and old individuals (70–86 years old) (Dungan et al., 2020). Interestingly, young obese individuals (21–24 years old, BMI: 34–46) have higher γH2AX expression in myonuclei than young lean individuals (21–24 years old, BMI: 20–25) (Dungan et al., 2020). These studies suggested that aging and obesity, which are related to the impairment of muscle function, regenerative capacity, and muscle volume, tend to increase the abundance of senescent muscle cells; however, this idea remains still controversial.

### Senescence of Muscle Stem Cells

**FIGURE 1:**
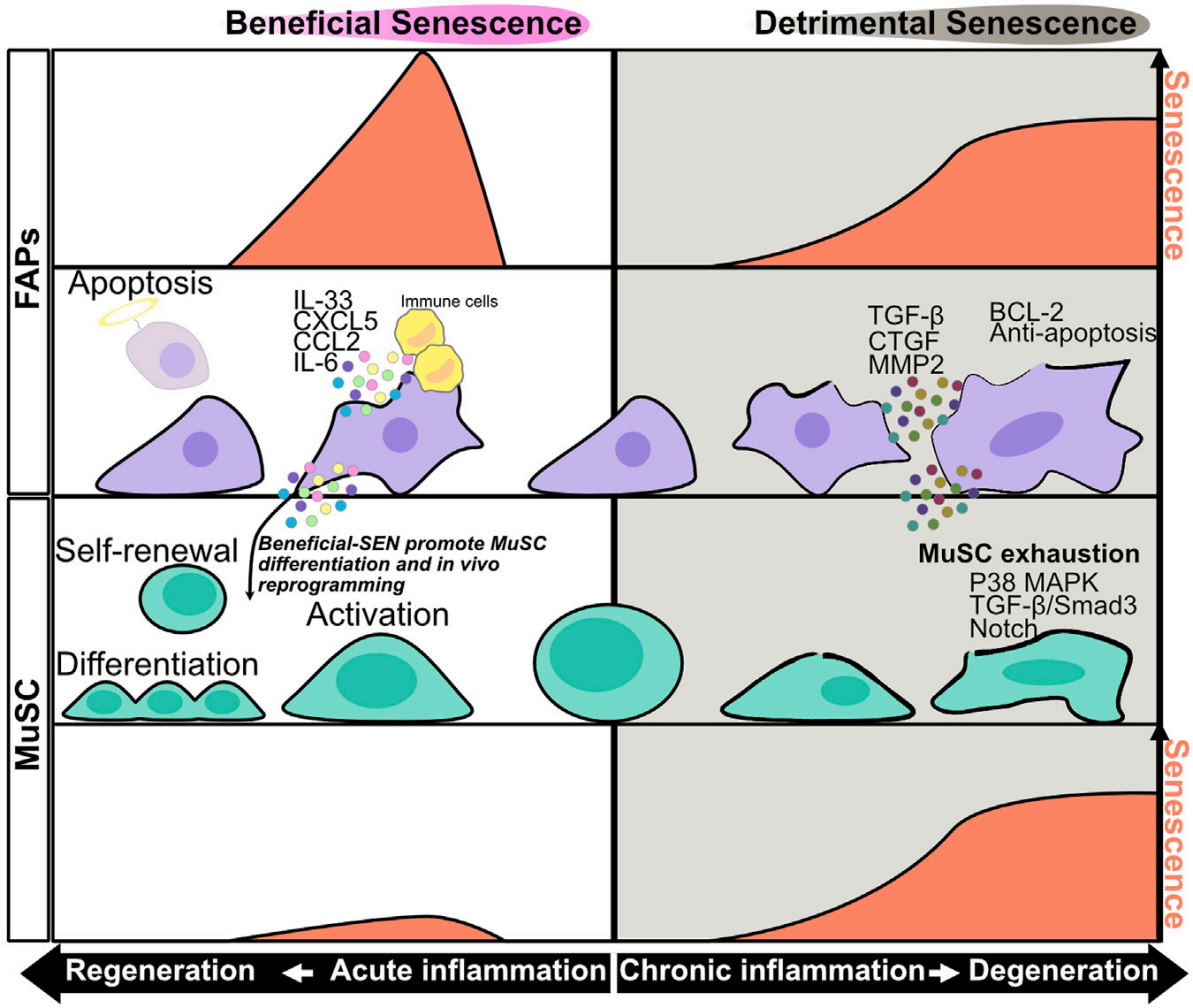
Senescence in muscle stem cells and fibro/adipogenic progenitors during acute and chronic inflammation. During acute inflammation leading to regeneration, the level of senescence in fibro/adipogenic progenitors (FAPs) transiently increases at the early phase of acute inflammation and decreases as the cell approaches regeneration. SASP factors produced during acute inflammation promote immune cell recruitment and muscle stem cell (MuSC) activation. IL-6 has the potential to promote *in vivo* reprogramming of MuSCs. The expression level of senescence decreases after clearance of senescent FAPs by immune cells. During chronic inflammation leading to degeneration phase, the levels of senescence in FAPs and MuSCs gradually increase as the cell approaches degeneration. SASP factors produced during chronic inflammation, especially TGF-β, promote MuSC senescence and exhaustion, resulting in impairment of muscle regeneration impairment. Chronically senescent FAPs have an anti-apoptotic phenotype, and their prolonged accumulation in muscle results in fibrosis.

The mechanism of sarcopenia is not fully understood, but some studies have implicated that senescence of muscle stem cells (MuSCs), also known as satellite cells, in this process (Carlson et al., 2008; Cosgrove et al., 2014; Sousa-Victor et al., 2014; García-Prat et al., 2016). In geriatric mice, MuSCs express high levels of SA-β-Gal and *Cdkn2a*, *Cdkn2b*, and *Igfbp5* mRNA, resulting in impaired muscle regeneration after injury (Sousa-Victor et al., 2014). In addition, transplantation of geriatric mouse–derived MuSCs decreased regenerative capacity after injury, even in young mice (Cosgrove et al., 2014). These results indicate that cellular senescence in MuSCs impairs muscle regeneration (Figure 1). A potential mechanism of senescence-induced impairment of muscle regeneration involves premature senescence induced by persistent p38 MAPK activity and stem cell exhaustion (Figure 1) (Bernet et al., 2014; Cosgrove et al., 2014; Sousa-Victor et al., 2014; Blau et al., 2015). Another study showed that Slug, a member of zinc-finger transcriptional factor in the Slug/Snail superfamily, is downregulated with aging; MuSC-specific knockout of Slug promotes p16^INK4a^ expression in MuSC after muscle injury, resulting in impaired muscle regeneration (Zhu et al., 2019). Similarly, the cell surface protein Cdon, which positively regulates myogenesis, is downregulated in progeria model mice, and MuSC-specific knockout of Cdon promotes γH2AX expression in MuSCs after injury, resulting in impaired muscle regeneration (Bae et al., 2020). In addition to aging, obesity and muscular dystrophy are associated with accumulation of senescent MuSCs (Kudryashova et al., 2012; Mu et al., 2015; Roux et al., 2015; Zhang et al., 2016; Dungan et al., 2020; Sugihara et al., 2020). Although the disease-specific mechanism of senescence induction remains unknown, senescence in MuSCs can result from reduced mitophagy, TGF-β–induced Smad3 activation, and over-activation of Notch (Figure 1) (Carlson et al., 2008; Mu et al., 2015; García-Prat et al., 2016).

### Senescence of Fibro/Adipogenic Progenitors

**FIGURE 2:**
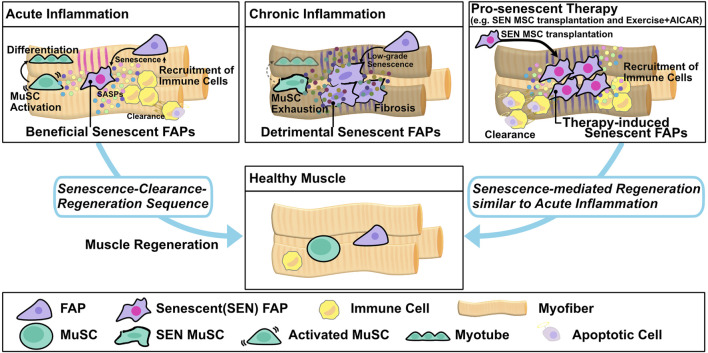
Muscle regeneration by the senescence–clearance–regeneration sequence. Fibro/adipogenic progenitors (FAPs) act as beneficial senescent cells and express high levels of senescence factors during acute inflammation. Beneficial senescent cells secrete SASP factors to recruit immune cells and promote muscle stem cell (MuSC) activation and differentiation, and then the senescent FAPs are eliminated by immune cells to complete tissue regeneration (senescence–clearance–regeneration sequence). On the other hand, in chronic inflammation, the FAPs produce lower levels of senescence factors than in acute inflammation and act as detrimental senescent cells. Pro-senescence therapy, e.g., transplantation of senescent mesenchymal stromal cells (MSC) or the combination of exercise and AICAR, induces resident FAP senescence and promotes immune recruitment. Subsequently, the FAPs are cleared by immune cells, and muscle regeneration is completed via the senescence–clearance–regeneration sequence, as seen in acute inflammation.

Muscle resident mesenchymal progenitors, known as fibro/adipogenic progenitors (FAPs), contribute to muscle regeneration under physiological conditions and to ectopic tissue formation under pathological conditions (Joe et al., 2010; Uezumi et al., 2010; Murphy et al., 2011; Uezumi et al., 2011; Ito et al., 2013; Uezumi et al., 2014; Lemos et al., 2015; Scott et al., 2019; Theret et al., 2021). Upon injury, under conditions of acute inflammation, the number of FAPs increases transiently from days 2–5 and then returns to basal levels 14–21 days after injury to complete muscle regeneration; however, in chronic inflammation, prolonged FAP proliferation and deficient clearance results in FAP accumulation and fibrosis (Joe et al., 2010; Uezumi et al., 2010, 2011, 2014; Murphy et al., 2011; Ito et al., 2013; Lemos et al., 2015; Scott et al., 2019). These dynamic changes in FAP proliferation and clearance might be important for regulation of muscle regeneration and degeneration, but the underlying mechanisms are not fully understood. In regard to FAP proliferation, a recent study showed that expression of hypermethylated in cancer 1 (Hic1) maintains FAPs in a quiescent state, whereas reduced expression of Hic1 immediately after injury results in FAP proliferation (Scott et al., 2019). Another study suggested that activation of the IL-4/STAT6 pathway promotes FAP proliferation (Heredia et al., 2013), and that IL-15 also promotes FAP proliferation. In regard to FAP clearance, Ly6C + TNF-α–rich macrophages play an important role in FAP clearance via their pro-apoptotic effects (Lemos et al., 2015). Moreover, senescence in FAPs after acute muscle injury promotes SASP expression and recruitment of phagocytic cells to promote FAP clearance (Figure 1) (Chikenji et al., 2019; Saito et al., 2020). On the other hand, FAP clearance is impaired by the anti-apoptotic phenotype of these cells, e.g., excessive TGF-β signaling by Ly6C- macrophages activates pro-survival signaling in FAP (Figure 1) (Lemos et al., 2015; Juban et al., 2018; Saito et al., 2020). Collectively, these studies indicate that pro-inflammatory, pro-apoptotic/anti-inflammatory, anti-apoptotic signals must be balanced in order to achieve complete muscle regeneration. In a mouse model of chronic inflammatory myopathy, senescent FAPs promote the recruitment of macrophages and NK cells and activate MuSCs, resulting in muscle regeneration (senescence–clearance–regeneration sequence) (Figure 2) (Chikenji et al., 2019). Furthermore, during acute muscle injury, senescent FAPs increase the expression of several cytokines, and IL-33 expression levels in FAPs are correlated with *Cdkn2a* and *Trp53* expression levels (Saito et al., 2020). IL-33 is a potent inducer of pro-inflammatory cytokines and chemokines, promotes the production of TNF-α by macrophages (Liew et al., 2010; Xu et al., 2017), and also regulates muscle regulatory T cells (Tregs) that promote muscle regeneration (Kuswanto et al., 2016). Thus, senescent FAPs can activate immune cells and MuSCs to create a state of regenerative inflammation (Figure 2). In a mouse model of early aging, BubR1H/H, p16^INK4a^ expression in FAPs is upregulated, and muscle regeneration after injury is delayed (Baker et al., 2013). Interestingly, mouse knockouts of two other senescence-related genes, *p53* and *p21* (BubR1H/H; p53-/- and BubR1H/H; p21-/-) impairs the muscle regeneration capacity of the BubR1H/H mice (Baker et al., 2013). Another study reported that senescent FAPs are more abundant in obese humans and a rat model of Duchenne muscular dystrophy; however, the causal link between the increased senescent FAPs and muscle pathology remains unclear (Dungan et al., 2020; Sugihara et al., 2020).

### Senescence-Associated Reprograming

As mentioned above, cellular senescence in MuSCs increases with aging and disease, which adversely affects skeletal muscle. By contrast, senescence in FAPs not only increases with aging and disease but also contributes to muscle regeneration. It remains controversial whether senescent cells in skeletal muscle inhibit or promote muscle regeneration; however, senescent cells may be able to induce MuSCs reprogramming in response to muscle injury. One study used i4F mice to determine whether senescent cells affect MuSC reprogramming (Chiche et al., 2017). When treated with doxycycline, i4F mice induce expression of four mouse reprogramming genes, *Oct4*, *Klf4, Sox2*, and *c-Myc* (OSKM, known as the Yamanaka factors) (Mosteiro et al., 2016; Chiche et al., 2017). The results revealed that muscle injury promoted *in vivo* reprogramming in muscle; interestingly, the Nanog + reprogrammed cells were frequently near senescent cells located in the interstitial space (Chiche et al., 2017). Furthermore, when senescent cells were depleted, or production of the SASP factor IL-6 was suppressed, the reprogramming efficiency of MuSCs was reduced, indicating that senescent cells, which become more abundant in response to injury, may contribute to muscle regeneration by promoting MuSC reprogramming (Figure 1) (Chiche et al., 2017). Another study supported the idea that the senescence-related factor p21 is important for *in vivo* reprogramming mediated by muscle regeneration (Wang et al., 2021) by showing that myofiber-specific short-term induction of OSKM promotes muscle regeneration and upregulation of p21, which attenuates Wnt4 signaling (Wang et al., 2021). These findings could have implications for the development of novel therapeutic strategies based on transient induction of senescence and muscle-lineage cell reprogramming.

## The Effect of Immunosenescence on Muscle

Immunosenescence is an age-related process of immune dysfunction that contributes to morbidity and mortality (Kennedy et al., 2014; Ventura et al., 2017; Duggal et al., 2019). Among several biomarkers for immunosenescence that have been reported, p16^INK4a^ is a reliable marker for senescence in T cells, B cells, and macrophages (Liu et al., 2009; Cudejko et al., 2011; Liu et al., 2011; Liu et al., 2019). In T cells, expression of the proliferation marker Ki-67 and activated T cell marker are observed with aging, and depletion of p16^INK4a^ attenuates these features of senescence (Liu et al., 2009). The authors of the same study showed that depletion of p16^INK4a^ in B cells attenuates senescence hallmarks that arise with aging (Liu et al., 2009). Bone marrow–derived macrophages isolated from p16^INK4a^-deficient mice downregulate genes associated with inflammatory M1 macrophages and increase expression of genes associated with M2 macrophages (Cudejko et al., 2011). The expression level of the p16^INK4a^ in macrophages does not affect M1/M2 polarization, although p16^INK4a^-high macrophages have higher phagocytic activity than p16^INK4a^-low macrophages (Liu et al., 2019). Skeletal muscle regeneration is regulated not only by MuSCs and FAPs but also by immune cells; hence, immunosenescence must affect muscle regeneration, although evidence of a connection between the two phenomena remains limited. Recently, a study using Vav-iCre(+/−); Ercc1 (−/fl) mice showed that immune cell–specific induction of senescence decreased muscle strength and impaired regeneration after muscle injury (Yousefzadeh et al., 2021). Furthermore, immune cell–specific senescence induction increased the number of infiltrating macrophages and decreased the ratio of M2 to M1 macrophages after muscle injury (Yousefzadeh et al., 2021). On the other hand, the Newcastle 85 + Study, a prospective, population-based study of very old adults living in the Newcastle and Tyneside regions, United Kingdom, showed that immunosenescence profiles were not associated with muscle function and sarcopenia risk (Granic et al., 2020). Although it remains controversial whether immunosenescence affects muscle inflammation and regeneration, a deeper understanding of the mechanistic regulation of immunosenescence and muscle regeneration could help promote the progress of senotherapy for muscle aging and disease.

## Potential of Senotherapy for Muscle Inflammation and Regeneration

Proper regulation of two types of senescent cells, detrimental-senescent cells and beneficial-senescent cells, could exert beneficial effects on muscle regeneration. Several previous studies showed that senolytics restore muscle loss and inflammation in aged mice. Upon genetic elimination of senescent cells using INK-ATTAC mice, mRNA expression of *p16*, *p21*, *p19*, and SASP genes including *Pai1*, *Il6*, *Mmp6*, and *Mmp13* were reduced in aged C57BL/6 mice and BubR1H/H mice, a model of early aging (Baker et al., 2011, 2016). Pharmacological elimination of senescent cells by dasatinib/quercetin treatment also decreased muscle strength and function in aged C57BL/6 mice, which develop age-related muscle loss and inflammation in skeletal muscle (Xu et al., 2018). Another senolytic drug, ABT263, also inhibits production of the SASP factors IL-6, TGF-β, and IL-1β in a rat model of Duchenne muscular dystrophy (Sugihara et al., 2020). Treatment with the NAD + precursor nicotinamide riboside (NR) prevents MuSC senescence and decreases production of SASP factors in aged mice as well as in *mdx* mice, a model of muscular dystrophy (Zhang et al., 2016). On the other hand, other studies suggested that pro-senescence therapy can promote muscle regeneration. The combination of exercise and AICAR, a cell-permeable AMPK activator, promotes muscle regeneration by inducing FAP senescence in chronic inflammatory myopathy model mice (Wang et al., 2003; Saito et al., 2020). Another study found that transplantation of functional senescent mesenchymal stromal cells (MSC) treated with placenta extract promoted muscle regeneration in chronic inflammatory myopathy model mice (Figure 2) (Chikenji et al., 2019). In mechanistic terms, transplantation of functional senescent MSC, which have phenotypes distinct from those of cells induced to senesce by continuous cultivation, promotes FAP senescence followed by phagocytic cell recruitment and MuSC proliferation (Figure 2) (Chikenji et al., 2019). In cardiac muscle, fibroblast senescence plays important roles in heart regeneration (Feng et al., 2019; Sarig et al., 2019). One study found that treatment with CCN1, a matricellular protein, induced PDGFRα+ fibroblast senescence, decreased cardiac fibrosis, and triggered the expression of SASP factors including IL-1a and IL-6, thereby promoting cardiomyocyte proliferation and heart regeneration (Feng et al., 2019). Interestingly, the senolytic drug ABT263 decreases cardiomyocyte proliferation and increases proliferation of PDGFRα+ fibroblasts, thus promoting heart fibrosis (Feng et al., 2019). Another study found that treatment with the extracellular matrix molecule agrin induced transient senescence in vimentin + fibroblasts and promoted heart regeneration after myocardial infarction (Sarig et al., 2019). Together, the results of these studies suggest that pro-senescent therapy, especially targeting fibroblasts or mesenchymal stromal cells, represents a novel strategy for regulating muscle inflammation and regeneration (Figure 2).

### Exercise-Mediated Cellular Senescence: Potential of Senotherapy

**FIGURE 3:**
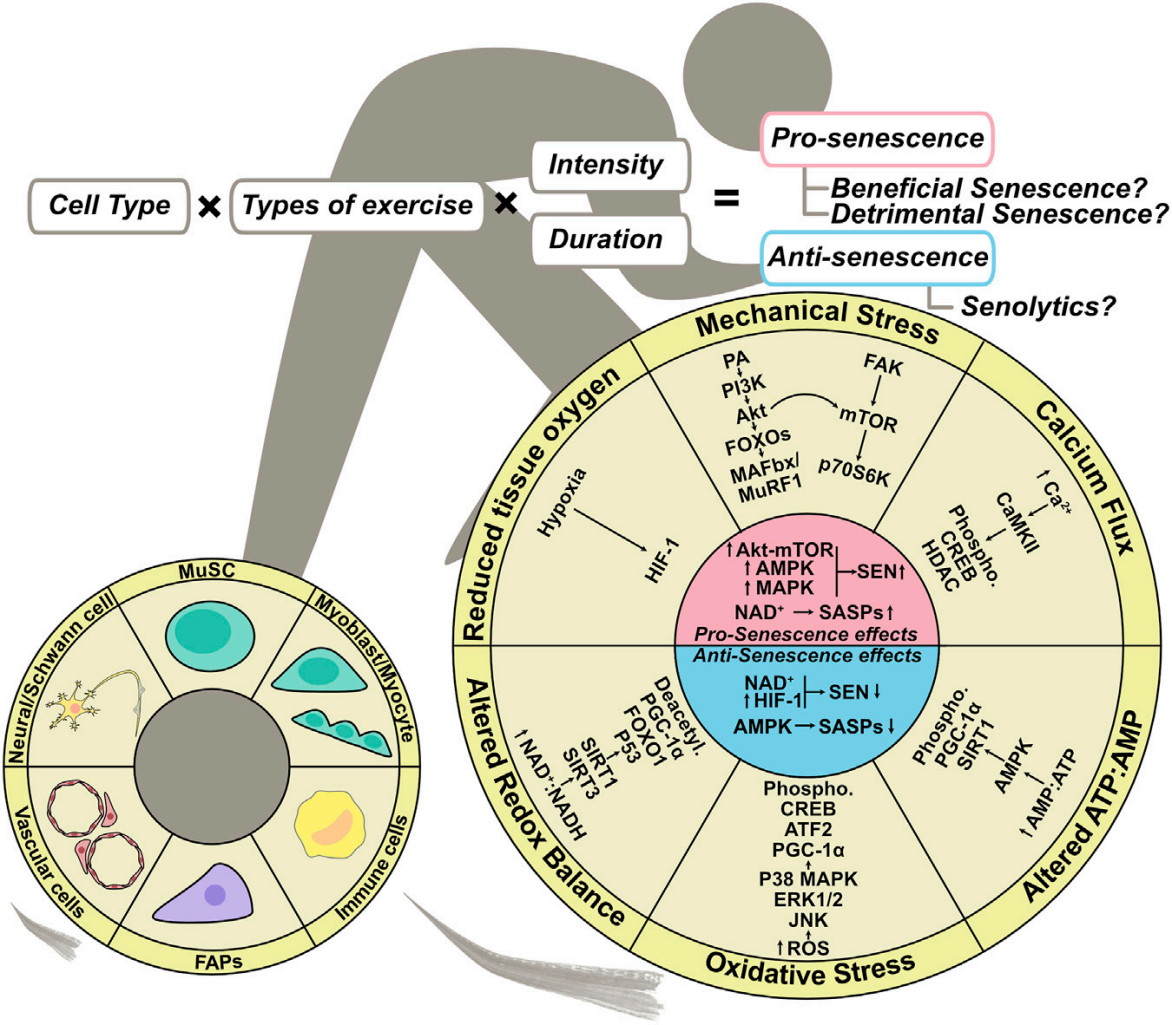
Exercise regulation of senescence in skeletal muscle. Exercise-induced complex stimulation if skeletal muscle can regulate senescence. Mechanical stress, altered ATP:AMP ratio, and oxidative stress–mediated up-regulation of Akt-mTOR, AMPK, and MAPK signaling promote senescence, whereas NAD + metabolism governs SASP expression. On the other hand, altered ATP:AMP ratio, altered redox balance, and AMPK activation mediated by reduced tissue oxygen inhibit SASP expression, and NAD + metabolism and HIF-1 activation inhibit senescence. The outcome of exercise-induced muscle regeneration could depend on cell type, exercise type, and the intensity and frequency, all of which affect senescence.

Continuous exercise is effective for maintaining homeostasis and improving muscle function by increasing muscle mass and oxygen supply. These effects of exercise are induced by a complex of stimuli on skeletal muscle, including mechanical stress, oxidative stress, changes in the AMP:ATP ratio, an increase in calcium flux, changes in redox balance, and a decrease in the partial pressure of intracellular oxygen (Figure 3) (Egan and Zierath, 2013). High-force muscle contractions caused by resistance training transiently disrupt the sarcolemma and increase the concentration of membrane phosphatidic acid (PA) by activating phospholipase D (PLD), which in turn activates PI3K, Akt, and mTOR, resulting in muscle protein synthesis (Fang et al., 2001). In addition, Akt signaling suppresses muscle RING finger 1 (MuRF1) and muscle atrophy F box (MAFbx) by inhibiting Forkhead box–containing proteins (FOXOs), resulting in the suppression of muscle protein degradation (Sandri et al., 2004; Egan and Zierath, 2013). Muscle contraction also activates focal adhesion kinase (FAK), a mechanosensor, to stimulate muscle protein synthesis in an mTOR-dependent or -independent manner (Wilkinson et al., 2008; Durieux et al., 2009; Klossner et al., 2009; Philp et al., 2011). The increase in calcium ion concentration caused by muscle contraction can induce the phosphorylation of calmodulin-dependent protein kinases II (CaMKII) (Rose and Hargreaves, 2003), which in turn induces phosphorylation and nucleo-cytoplasmic shuttling of HDAC4 (Liu et al., 2005). Exercise-induced mechanical stress can activate production of ROS, which stimulates the MAPK subfamilies ERK1/2, JNK, and p38 MAPK (Kramer and Goodyear, 2007). The metabolic changes that occur during muscle contraction affect the AMP:ATP and NAD+:NADH ratios, which induce activation of AMP-activated protein kinase (AMPK) and sirtuins (Sirt1, Sirt3), respectively (Jørgensen et al., 2006; Cantó et al., 2009; White and Schenk, 2012). Exercise also decreases the partial pressure of oxygen in skeletal muscle and activates HIF-1α (Ameln et al., 2005). Thus, exercise has the potential to regulate various signaling pathways, some of which are involved in the control of cellular senescence (Wang et al., 2003; Jones et al., 2005; Welford and Giaccia, 2011; Davalli et al., 2016; Zhang et al., 2016; Nacarelli et al., 2019; Chan et al., 2020; Vliet et al., 2021) (Figure 3). Accordingly, senescence-targeted exercise therapy has enormous potential for clinical benefit.

A systematic review (Chen et al., 2021) discussed whether exercise has a senolytic effect on various types of cells and tissues, as demonstrated by human and animal studies. The authors reported the senolytic effect on human skeletal muscle (Wu et al., 2019), human skeletal muscle–derived vascular endothelial progenitor cells (Yang et al., 2018), rat skeletal muscle (Fan et al., 2014), mouse skeletal muscle (Yoon et al., 2019), and mouse skeletal muscle–derived FAPs. Three studies using whole skeletal muscle tissue reported no change in the number of senescent cells or the expression of p16^INK4a^ and p21 protein after exercise (Fan et al., 2014; Wu et al., 2019; Yoon et al., 2019). A study by Yang et al. on vascular endothelial progenitor cells derived from human skeletal muscle reported a reduction in senescent vascular endothelial progenitor cells following squat training in which resistance was set at 70% of each individual’s one-repetition maximum (1RM) (Yang et al., 2018). On the other hand, cellular senescence of FAPs was induced by downhill running at an intensity that caused muscle damage (Saito et al., 2020). The exercise-induced senescent FAPs exhibited a pro-apoptotic phenotype with elevated expression of SASP factors, similar to the FAP phenotype during muscle regeneration after acute inflammation, indicating that FAPs may function as beneficial senescent cell (Saito et al., 2020). It remains unclear whether cellular senescence in skeletal muscle is induced or inhibited depending on the type of exercise, intensity, duration, and cell type, and whether the induced or eliminated senescent cells are beneficial or detrimental. However, regulation of cellular senescence by exercise represents a new therapeutic strategy as a senotherapy for skeletal muscle.

## Discussion

**FIGURE 4:**
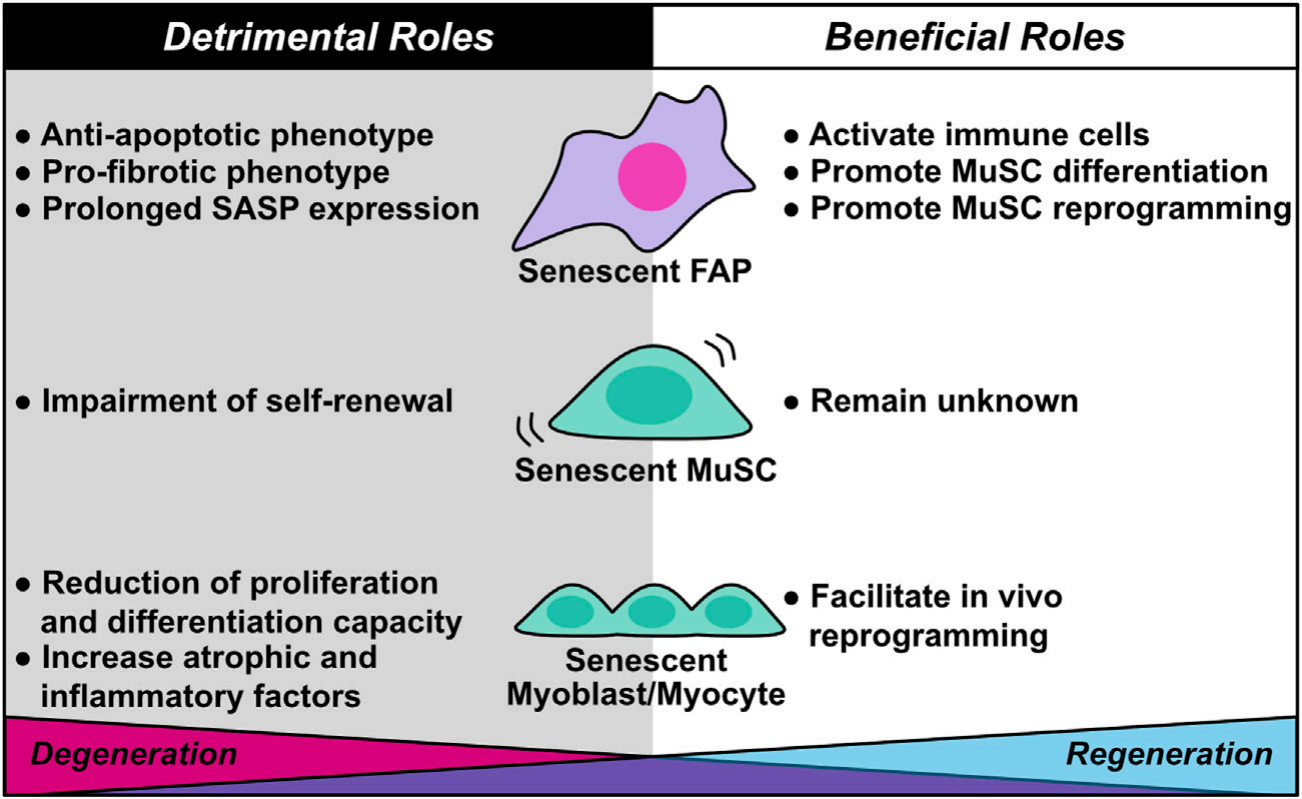
Diverse roles of cellular senescence in skeletal muscle regeneration and degeneration. Cellular senescence in skeletal muscle plays dual roles in muscle regeneration and degeneration. Although senescent fibro/adipogenic progenitors (FAPs) activate immune cells and promote muscle stem cell (MuSC) differentiation and reprogramming during muscle regeneration, they also contribute to muscle degeneration through anti-apoptotic and pro-fibrotic phenotypes, as well as prolonged SASP expression. Senescent MuSC exhibits impairment of self-renewal capacity, which induces stem cell exhaustion. The beneficial roles of senescent MuSC remain unknown. Senescent myoblasts/myocytes facilitate their own *in vivo* reprogramming, a beneficial role; however, senescent myoblasts/myocytes also contribute to muscle degeneration by decreasing proliferation and differentiation capacity and increasing expression of atrophic and inflammatory factors.

Understanding the mechanism of cellular senescence–mediated tissue regeneration and degeneration is essential for maintaining healthy skeletal muscle. Mounting evidence supports the hypothesis that cellular senescence in skeletal muscle plays diverse roles in muscle regeneration and degeneration. In this review, we described the basic mechanisms of cellular senescence and the beneficial and detrimental effects of senescence on muscle regeneration and degeneration (Figure 4). Unfortunately, there are no distinguishing features of beneficial and detrimental senescent cells; however, several pieces of evidence suggested that a transient increase in the proportion of senescent cells exerts beneficial effects, whereas prolonged accumulation of senescent cells exerts detrimental effects. In regard to PDGFRα+ mesenchymal cells in skeletal muscle and cardiac muscle, induction of senescence is likely to have a positive effect on muscle regeneration by promoting the proliferation of parenchymal cells and inhibiting fibrosis by activating phagocytic cells.

A deeper understanding of the relationship between cellular senescence and muscle physiology and pathology will lead to advances in research on skeletal muscle. However, there are still some outstanding questions. First, what are the molecular and cellular differences between beneficial senescence and detrimental senescence? Second, which cell types, such as MuSCs and FAPs, frequently enter senescent states during acute injury, chronic muscle disease, and aging? Third, what kind of SASP factors contribute to muscle regeneration or chronic inflammation. Fourth, can senotherapy attenuate chronic muscle inflammation and promote muscle regeneration? In addition, which type of senotherapy (e.g., senolytics, senomorphics, or pro-senescence therapies) can exert positive effects on muscle health? Fifth, can exercise-mediated senescence regulation provide a therapeutic effect against chronic muscle diseases or aging? If so, how can we optimize the intensity and frequency of exercise to regulate senescence in each cell type? Addressing these questions will lead to the development of therapies that target cellular senescence.

Finally, many senotherapy drugs have been developed; some have proceeded to clinical trials, and even newer senotherapy drugs continue to be reported today. Undoubtedly, senotherapies, including not only pharmacological therapies, but also cell-based therapies, exercise therapies, and a combination of these therapeutic approaches could provide a beneficial outcome in patients with chronic inflammatory muscular disease and aging-related muscle dysfunction. Importantly, an in-depth understanding of the complex roles of senescent cells, which remain largely unknown, is essential for the development of truly effective senescence-targeting therapies.
